# The Role of Propagule Pressure, Genetic Diversity and Microsite Availability for *Senecio vernalis* Invasion

**DOI:** 10.1371/journal.pone.0057029

**Published:** 2013-02-20

**Authors:** Alexandra Erfmeier, Lydia Hantsch, Helge Bruelheide

**Affiliations:** 1 Institute of Biology/Geobotany and Botanical Garden, Martin Luther University Halle-Wittenberg, Halle (Saale), Germany; 2 German Institute for Integrative Biodiversity Research (iDiv), Bio City, Leipzig, Germany; French National Institute for Agricultural Research (INRA), France

## Abstract

Genetic diversity is supposed to support the colonization success of expanding species, in particular in situations where microsite availability is constrained. Addressing the role of genetic diversity in plant invasion experimentally requires its manipulation independent of propagule pressure. To assess the relative importance of these components for the invasion of *Senecio vernalis*, we created propagule mixtures of four levels of genotype diversity by combining seeds across remote populations, across proximate populations, within single populations and within seed families. In a first container experiment with constant *Festuca rupicola* density as matrix, genotype diversity was crossed with three levels of seed density. In a second experiment, we tested for effects of establishment limitation and genotype diversity by manipulating *Festuca* densities. Increasing genetic diversity had no effects on abundance and biomass of *S. vernalis* but positively affected the proportion of large individuals to small individuals. Mixtures composed from proximate populations had a significantly higher proportion of large individuals than mixtures composed from within seed families only. High propagule pressure increased emergence and establishment of *S. vernalis* but had no effect on individual growth performance. Establishment was favoured in containers with *Festuca*, but performance of surviving seedlings was higher in open soil treatments. For *S. vernalis* invasion, we found a shift in driving factors from density dependence to effects of genetic diversity across life stages. While initial abundance was mostly linked to the amount of seed input, genetic diversity, in contrast, affected later stages of colonization probably via sampling effects and seemed to contribute to filtering the genotypes that finally grew up. In consequence, when disentangling the mechanistic relationships of genetic diversity, seed density and microsite limitation in colonization of invasive plants, a clear differentiation between initial emergence and subsequent survival to juvenile and adult stages is required.

## Introduction

Genetic diversity within species and populations has been proven to have profound impacts on ecosystems by affecting community structure, nutrient fluxes and productivity, thereby affecting biotic interactions, such as resistance to disturbance and invasions [1]–[3]. Accordingly, some studies point at the need to take within-species variation more explicitly into account for ecosystem functioning [4]–[6]. While, in recent years, studies on the role of genetic variation in biological invasions have shown that the maintenance of high genetic diversity is a feature of successful invasions [7]–[10], genetic diversity has rarely been experimentally addressed to explain invasiveness of species despite some evidence of both additive and non-additive effects of genetic diversity on colonization success [7], [11]–[13]. One recent example elucidating the role of genetic diversity for expanding species was provided by Crawford and Whitney [14], who could demonstrate positive effects of genetic diversity on attributes of colonization success of *Arabidopsis thaliana*, i.e. seedling emergence, biomass, flowering duration and reproduction. However, establishing empirical relationships between invasion success and genetic diversity is not sufficient to demonstrate a mechanistic link [3]. The assumed mechanistic effect of high genetic diversity, i.e. high intraspecific diversity among genotypes, should be expected to operate analogously to mechanisms of species diversity within plant communities [15], [16]. Thus, high within-population genetic diversity might enhance coexistence of individuals, since fitness might be positively influenced if individuals are functionally different. Such complementarity effects are based on optimized resource use if assessed at the community level [17] and can lead to increased overall productivity [18].

Biodiversity experiments in grasslands have shown that diversity effects may comprise complementarity effects, i.e. reduced competition due to niche partitioning [19], but also effects of facilitation [20], [21]. Furthermore, diverse mixtures also include the possibility of sampling effects, i.e. the increased probability of including highly productive species, and of selection effects, i.e. that particular productive species might become dominant over the time. The distinction of these effects at the below-species level, including populations and genotypes within populations, thus, constituting a hierarchical structure, would require a sophisticated and expanding design with all populations and genotypes grown in monocultures. However, general diversity effects and the identification of sampling effects might still be addressed: Hughes et al. [3], e.g., recently argued that one might expect impacts of different genotypes that are similar to those of different species, but there has been only sparse experimental evidence so far. One such a rare experimental example was provided by Vellend et al. [22] who tested effects of genotype diversity on dandelion (*Taraxacum officinale*) invasiveness. They found sampling effects, i.e. the presence or absence of a particular genotype identity, to be more important for explaining invasiveness of dandelions than genotypic diversity per se.

Establishing a mechanistic link of genetic diversity in invasions requires accounting for confounding variables, i.e. further factors that affect both genetic diversity and invasion success. Genetic diversity in invading populations is supposed to be induced by multiple introductions and, in particular, by the number of propagules released into a new range or habitat [23]–[25]. However, propagule pressure, in the following defined as the number of seeds or propagules released at a local scale, has a direct positive effect on invasiveness independent of genetic diversity because a higher number of individuals reduce chance effects, e.g. induced by climate or biotic interactions [26], [27]. However, apart from effects of environmental stochasticity, chance effects, in addition, may negatively affect dynamics of biological invasions or reduce establishment and spread [28], [29]. Thus, when controlling for negative effects of density dependence [30], propagule pressure has been found to be a suitable predictor of invasion success [31]. In addition, a high number of propagules can be also expected to increase genetic diversity by conferring increased trait variability, and thus, accordingly, can indirectly impact a species' invasion success 32,33. However, high propagule pressure and high genetic diversity are not necessarily ultimately linked during plant species invasions. Disentangling these effects is not trivial, as increased propagule pressure in the field will always be connected to increased genetic diversity as long as an increasing number of different sources is involved [27]. Thus, the challenge is to manipulate genetic diversity at a constant level of propagule pressure.

Apart from seed availability, the availability of sites suitable for seedling establishment is another major determinant of recruitment success in plant populations [34]–[36]. During initial phases of colonization, expanding populations, in general, encounter a variety of unpredictable heterogeneous environments [37], [38], where the ability to respond adequately to new selection pressures is crucial. High genetic variation at the population level might, then, be particularly advantageous by offering a diverse set of genotypes that increases the probability of habitat match. Habitat characteristics associated with disturbance, eutrophication and open soil largely affect invasion, since all these processes result in increased levels of resources [39] and, thus, contribute to overcoming the constraints of establishment limitation. The results of a meta-analysis on seed addition experiments have shown, accordingly, that seed limitation, in particular, was more crucial in disturbed plots [40]. Thus, habitat or community susceptibility is supposed to interact with genetic diversity of colonizers and, accordingly, must be seen as an additional feature governing invasion success [41].

In the present study, we aim at quantifying the role of genetic diversity during invasions in interaction with propagule pressure at the local scale and microsite availability. *Senecio vernalis* Waldst. & Kit. is a suitable study organism to approach these questions experimentally. *Senecio vernalis*, spring groundsel, is an insect pollinated therophyte, native to West Asia and expanding to northern, central and Western Europe along railways [42], [43]. As ruderal species of the Asteraceae, *S. vernalis* is characterized by the release of large amounts of achenes per individual [42], [44] and therefore able of exerting high propagule pressure. Both the species' capability of producing high numbers of small seeds and the wide range of suitable habitat conditions for establishment accomplish the basic requirement for the colonization success of *S. vernalis* in Central Europe. Further investigations have provided evidence that populations of *S. vernalis* in Eastern Germany differ significantly in seed traits and germination characteristics (Hantsch et al., unpubl. data).

We established two fully randomised container experiments to analyse the relationship between 1) propagule pressure and genetic diversity as well as 2) microsite availability and genetic diversity for *S. vernalis*. In particular, we tested the hypotheses that i) high propagule pressure favours the establishment of *S. vernalis*, ii) high levels of microsite availability increase the establishment of *S. vernalis*. We, iii) hypothesized that, in both experiments, mixtures with higher levels of genetic diversity display higher establishment success than mixtures with lower genetic diversity. Furthermore, the effect of increased genetic diversity was expected to display interaction effects with propagule pressure and microsite availability, and to be more prominent at low levels of propagule density and microsite availability.

## Materials and Methods

### Ethics statement

No specific permissions were required for sampling activities, for we sampled only material at publicly accessible locations. We confirm that these locations are not privately-owned or protected in any way. The field studies did not involve endangered or protected species.

### Experimental set up

We created seed mixtures of different levels of genetic diversity based on the assumption that genetic diversity increases with increasing geographic distance. We expected lowest levels of genetic diversity within seed families (i.e. seeds sharing a common mother plant), intermediate levels within single populations (i.e. seeds of several mother plants), and highest levels of genetic diversity within mixtures of seeds sampled across several populations. For the same populations as in the current study, phenotypic variation and genetic diversity was studied as a function of habitat type by Hantsch et al. (unpubl. data). The authors found overall very high phenotypic and genetic variation and only little evidence of habitat type-dependent differentiation. In particular, there was a strong differentiation among populations in germination traits. We created four levels of seed mixtures of increasing diversity in origin and increasing distance labelled *seed families* (SF), *within populations* (WP), *proximate populations* (PP) and remote *populations* (RP). As a population pool, we referred to 18 populations located in Halle (Saale), Saxony Anhalt, Germany, and in its urban periphery, with a maximum distance of 27 km and a minimum distance of 0.22 km to each other (Table S1, Fig. S1). All populations were typical ruderal stands either assigned to roadside situations, semi-dry grasslands, stone quarries or soil dumps and were sampled from end of May to mid of June 2008. We collected achenes by seed families, as well as leaf material, exclusively for each of 20 individuals per population for creating seed mixtures of the seed family level (SF). For population based diversity levels (WP, PP, RP), we additionally collected achenes across 20 individuals per population if available and pooled them as a whole population's sample. This additional approach was necessary since the amount of seeds collected by seed family was not sufficient for compiling all mixtures and additional germination experiments. The seed material was stored at ambient temperature for about two months to allow for after-ripening. In the following, only ripe seeds displaying a hard coat with green-brown colouration were used for experimental treatments.

For the two container experiments, we set up six population mixtures each for *proximate* (PP) and *remote population* (RP). Each of the mixture replicates was composed of five populations. For the level of *remote population* (RP), each one initial population out of the total pool of 18 populations was selected at random. Subsequent filling of populations was done by random selection of additional four populations out of the sub-pool of those populations with a minimum distance of >13 km to the respective initial population. Accordingly, seed mixtures of *proximate populations* (PP) referred to eight populations of the total population pool (Pop3, Pop4, Pop5, Pop6, Pop8, Pop9, Pop10 and Pop14). This restricted pool included populations located in the central part of the total study area, thus, representing the nested core area with a population distance <7 km to each other.

For the *within population* level (WP), we included achenes sampled across several individuals within populations. A total of 12 populations was randomly selected as replicates. For the *seed family* level (SF), we created a total of 18 replicates, referring to each of three populations from the groups of *proximate* and *remote populations* with each three individuals within population as replicates. The complete set of all treatments, thus, comprised a total of 42 mixtures. For details on exact composition of seed mixtures by populations and individuals see Supplementary Data (Table S2). The two experiments were conducted independently. Random assignment of populations to population mixtures resulted in a nearly complete overlap between the two experiments for populations used in the mixtures of *proximate population* (PP). As a consequence of random assignment from a larger population pool for the mixtures of *remote populations* (RP), the overlap between the experiments was much smaller with each three populations being exclusively assigned to RP mixtures of the seed addition experiment (Pop1, Pop5, Pop13) and the microsite experiment (Pop3, Pop4, Pop6), respectively (see Table S2).

We used germination data available for all populations from Hantsch et al. (unpubl. data) and calculated mean expected germination of *S. vernalis* for the diversity levels *proximate populations* (PP) and *remote populations* (RP) separately by experiment (see Table S2). In a two-factorial ANOVA, population mixtures differed in the calculated expected germination between the two diversity levels applied (p = 0.020), with higher expected germination in mixtures of *proximate population* (PP) than in the mixtures of *remote populations* (RP, Fig. S2).

### Container experiment I – Propagule pressure and genetic diversity

In the first experiment, we studied the role of propagule pressure, i.e. seed density, and genetic diversity for establishment of *S. vernalis*. We used containers of 20 cm diameter and a volume of 6 l filled with a substrate mixture composed by compost and sand with a ratio of 2∶1. Each container was planted with seven individuals of *Festuca rupicola* Heuff (Poaceae) that served as constant matrix in this experiment. *Festuca rupicola* individuals were raised by seeds from seed material collected in the neighbourhood of *S. vernalis* populations and planted as three week old seedlings a few days prior to the experiment. For seed addition of *S. vernalis*, we applied three levels of seed densities with 30, 60 and 90 achenes for each of the 42 seed mixtures. The intermediate level of seed number corresponded to a density of 2000 seeds m^−2^ following Turnbull et al. [45]. In addition, we both reduced and increased density each by 50%. Each density level comprised exact replicates of the assigned mixtures of genetic diversity, yielding a total of 126 containers.

All containers were positioned randomly in one experimental plot in the Botanical Garden of the Institute of Biology/Geobotany (51.488889°N, 11.961389°E) and regularly watered to ensure optimal water availability during the experiment. The experiment ran for 15 weeks from May to September 2009.

### Container experiment II - Microsite availability and genetic diversity

A second experiment was designed for analysing effects of microsite availability and genetic diversity of propagules on establishment and growing success of *S. vernalis* early life-stage development. For this approach, we proceeded as in the first experiment but manipulated the matrix density of *F. rupicola* while keeping the seed density level constant at the intermediate level of 60 seeds per container. We created three levels of microsite availability by planting 5, 10 or 15 *Festuca* individuals per pot. In addition, we used matrix-free containers as control pots for open soil establishment. While all seed mixtures applied were anew chosen at random as described above, mixtures of genetic diversity levels were exactly repeated across all four matrix density levels. Container experiment II lasted 12 weeks from June to September 2009 and included 168 pots.

### Data collection

In both experiments, during the first four weeks, monitoring occurred at weekly intervals, but with increasing monitoring interval lengths thereafter. At each monitoring date, we assessed the total abundance of *S. vernalis* individuals in each container. In the following we refer to the number of individuals after 1 week and after 12 weeks as “initial abundance” and “establishment”, respectively. Maximum abundance per container was assessed across all monitoring dates. In addition, individuals encountered were assigned to size classes to quantify the demographical development of individuals over time. Individuals were classified either as seedling (stage 1, with cotyledons only), as small individuals (stage 2, with a length of largest leaf <1 cm), or as larger individuals (stage 3, with a minimum of five leaves, the largest one among them with a length of >1 cm). We calculated log ratios of size classes to assess the demographical development of *S. vernalis* during the experiment. Log size ratios were calculated as the logarithm to the base 10 of the proportion of summed individuals of size classes 2 and 3, divided by size class 1, i.e. the relative proportion of large individuals to small individuals (at the seedling stage). The more positive the log size ratio, the higher the abundance of large individuals compared to small individuals. For calculating log size ratios all values were added on by 1 to avoid division by zero.

At the end of the experiments, containers of both studies were harvested. *S. vernalis* samples were dried for 48 h at 60°C for dry mass determination to yield overall productivity of mixtures.

### Data analysis

Statistical analyses were done with SAS 9.2 [46]. All data were tested for normal distribution. Calculated log size ratio was the only variable that displayed normal distribution. In order to appropriately deal with the skewness of the data, log10 transformation of abundance- and mass-related data was found to be the most adequate way to achieve normality. For transformation prior to analyses, all abundance data were added on by 1 to enable log-transformation in case of zero abundance. We applied a generalised linear mixed model (*proc GLIMMIX*), using Gaussian error distribution and identity link functions for both data sets and included the class variables *diversity* and *seed density* in the first container experiment and *diversity* and *microsite availability* in the second experiment as fixed factors. Calculated expected germination of a particular seed mixture applied was included as covariate into the model to account for genotype identity effects on maximum abundance calculated across all monitoring dates. In addition, we tested for effects of presence/absence for those populations in the RP mixtures that were exclusively assigned to either the Container experiment I or II. We included Pop 1, Pop 5, Pop 13 and Pop 3, Pop 4, Pop 6 in the models testing for additional covariate effects in the seed density experiment and the microsite availability experiments, respectively.

We ran an additional analysis on abundance data of all monitoring dates including ‘time’ as additional random factor to analyse cumulative establishment success of *S. vernalis*. Covariation due to repeated measurements was modelled with the random statement in *proc GLIMMIX* [46], defining the containers as subjects and adapting the error matrix through the residual option. Following the AIC criterion, statistics suggested best fit by including time and time×seed density in the model.

The tests of fixed effects are based on type III SS. We are aware that with applying the same model to different response variables we performed multiple tests. However, we did not apply Bonferroni correction for controlling potential type I errors, given some debate on the conservatism of this correction method [47], [48]. As our study aimed at screening potential effects of genetic diversity, we consider it appropriate to present raw p values and acknowledge multiple testing in interpretation. Post-hoc tests were realized on least square means estimates adjusted with Scheffé multiple range tests for all pair-wise comparisons. All figures were generated with the program R 2.12.1.

## Results

### Container experiment I - Seed density and genetic diversity

**Figure 1:**
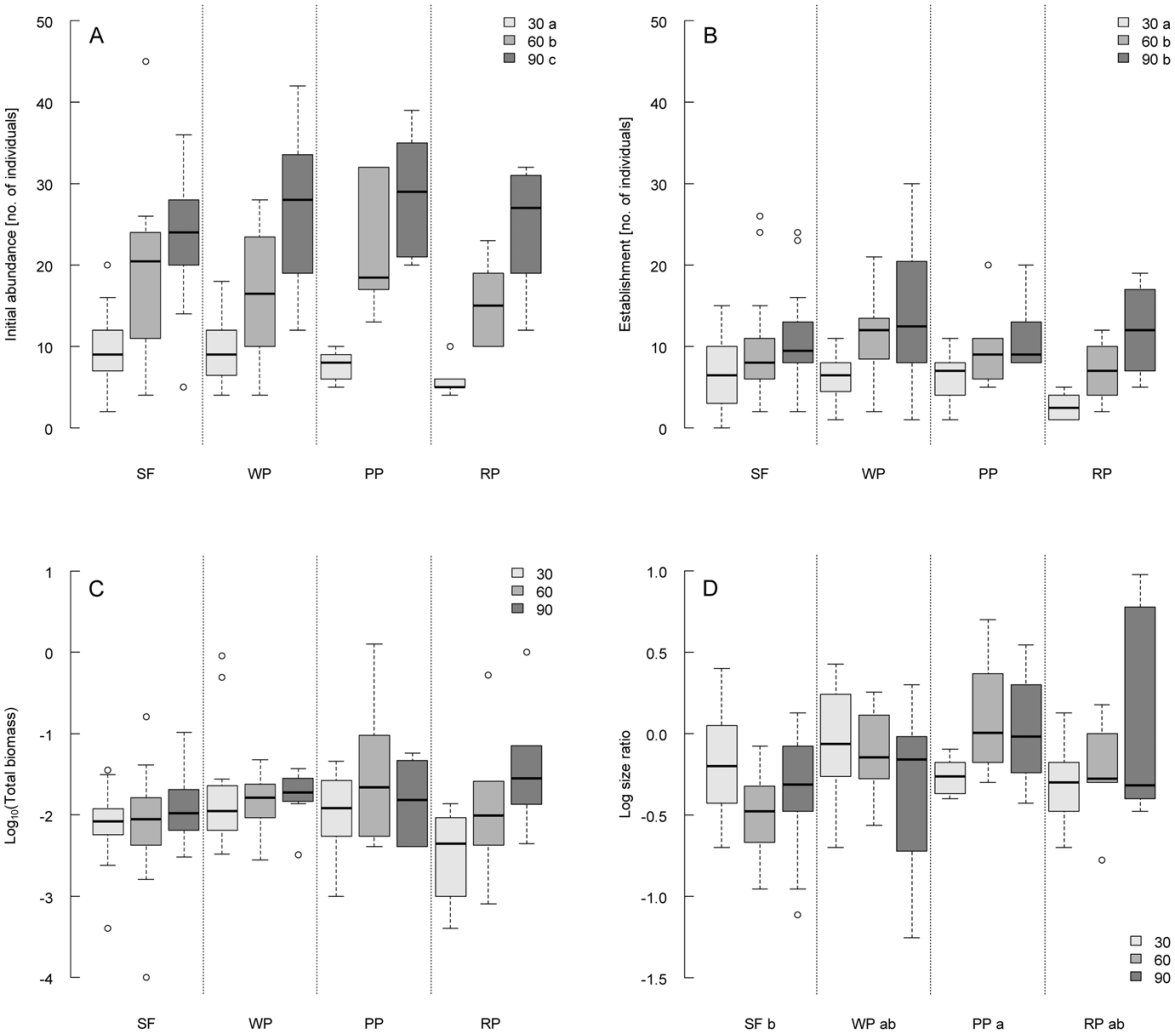
Experiment 1: Propagule pressure×genetic diversity. Box and Whisker plots of A) Initial abundance (week 1), B) Establishment (week 15), C) Total biomass (given as Log_10_ for reasons of clarity, week 15) and D) Log size ratio (week 15) of *Senecio vernalis* separately by genetic diversity levels applied: SF = *seed family* (n = 18 each), WP = *within population* (n = 12 each), PP = *proximate populations* (n = 6 each), RP = *remote populations* (n = 6 each). 30, 60, 90 = seed density levels applied. Different letters indicate significant differences between levels of main factors according to the Scheffé test. For further statistical details see Table 1.

Significant seed density effects were found for abundance of *Senecio vernalis* individuals (Table 1). Initial abundance of *S. vernalis* increased with increasing seed density level, with significant differences among all seed density levels (Table 1, Fig. 1A). Establishment after 15 weeks did not differ between the intermediate and high density levels, but was significantly higher than for the low level of seed addition (Fig. 1B). In contrast, total biomass and log size ratio of *S. vernalis* were not affected by seed density (Table 1, Fig. 1C, D).

**Table 1:** Experiment 1: Propagule pressure×genetic diversity.

Variable	Source of variation	df Num	df Den	F	p	Scheffé test
Initial abundance	Diversity	3	114	1.08	0.361	
[no. of individuals]	Seed density	2	114	57.35	**<0.001**	90>60>30
	Diversity×seed density	6	114	0.94	0.470	
Establishment	Diversity	3	114	1.48	0.223	
[no. of individuals]	Seed density	2	114	13.59	**<0.001**	90,60>30
	Diversity×seed density	6	114	0.60	0.730	
Log size ratio	Diversity	3	114	3.72	**0.013**	PP(>WP,RP)>SF
	Seed density	2	114	2.44	0.092	
	Diversity×seed density	6	114	1.88	0.090	
Total biomass	Diversity	3	110	1.89	0.136	
	Seed density	2	110	2.97	0.056	
	Diversity×seed density	6	110	1.86	0.094	

GLM for response variables of *Senecio vernalis*. Initial abundance reflects the number of *Senecio* individuals after 1 week. Establishment refers to the number of *Senecio* individuals after 15 weeks. Abundance and total biomass were log_10_(x+1)-transformed prior to analysis. Log size ratio gives the proportion of number of large individuals compared to those of small individuals as logarithm to the base 10. N = 126. The results of the Scheffé post hoc tests indicate the direction of significant differences between categories of each factor. 90, 60, 30 indicates seed density levels applied. Seed mixtures: RP = *remote populations*, PP = *proximate populations*, WP = *within population*, SF = *seed family*. The tests of fixed effects are based on type III SS, p values and degrees of freedom of numerator (df Num) and denominator (df Den) are shown. Bold numbers indicate significant effects (p<0.05).

**Figure 2:**
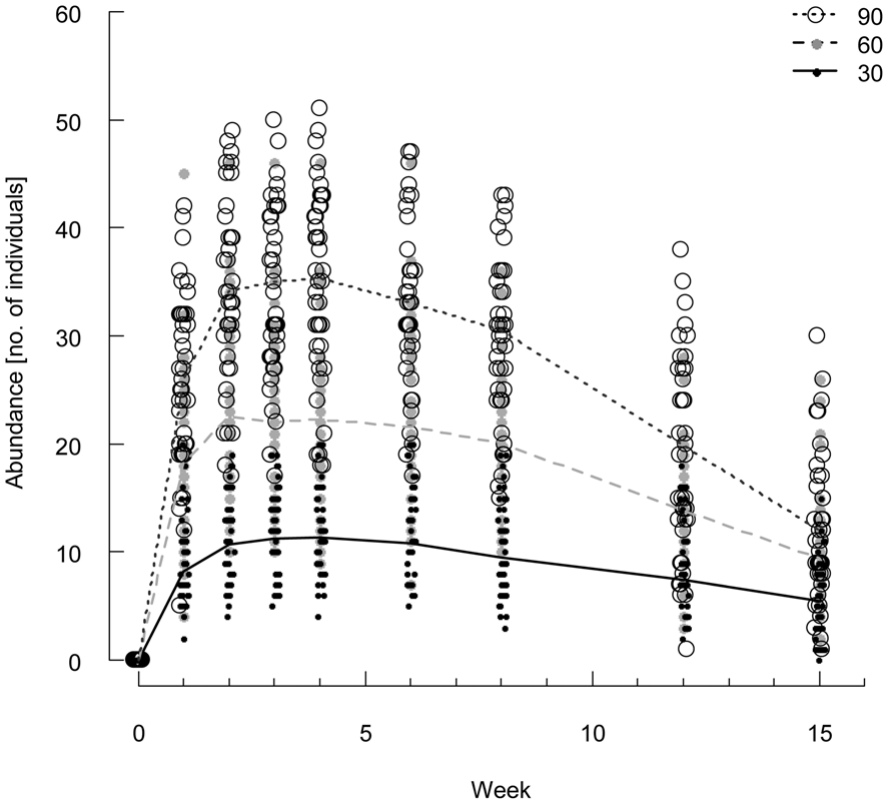
Experiment 1: Temporal development of *Senecio vernalis* abundance. Data are given separately for seed density levels applied. Open circles depict 90 seeds per container, filled grey circles depict 60 seeds per container, black dots show 30 seeds per container (n = 42 per seed level).

Abundance of *Senecio* individuals changed significantly with time. Beside a general decrease in abundance towards the end of the experiment (Fig. 2, Table S3), there was a significant effect of time and of the interaction between time and seed density as displayed by a more profound decrease in abundance within the set of highest (90 seeds) and intermediate (60 seeds) seed density levels than in the lowest seed density treatments (30 seeds) (Fig. 2). We accounted for covarying effects of the potential germination capacity of seed mixtures on maximum abundance by including calculated expected germination as covariate into the model; however, the test revealed no additional significant effect of the covariate (Table S4).

There were only a few effects of genetic diversity. Log size ratio was affected by genetic diversity (Table 1); mixtures of *proximate populations* (PP) displayed a more balanced and significantly higher proportion of large than of small individuals compared to seed applications from separate *seed families* (SF; Fig. 1D). Thus, with increasing levels of seed diversity we found a relative higher proportion of seedlings that reached the highest size class. This trend was not fully supported by the mixtures of *remote populations* (RP) which displayed an intermediate log size ratio. In addition, variation in initial abundance and establishment increased with increasing diversity levels as indicated by larger Whisker ranges in Fig. 1D.

There were no significant interaction effects between levels of seed density and genetic diversity in any of the variable studied.

Presence/absence of exclusive populations in the RP mixtures affected the overall results only marginally, but presence of single populations accounted for additional variation depending on the variable tested (Table S5).

### Container experiment II - Microsite availability and genetic diversity

**Figure 3:**
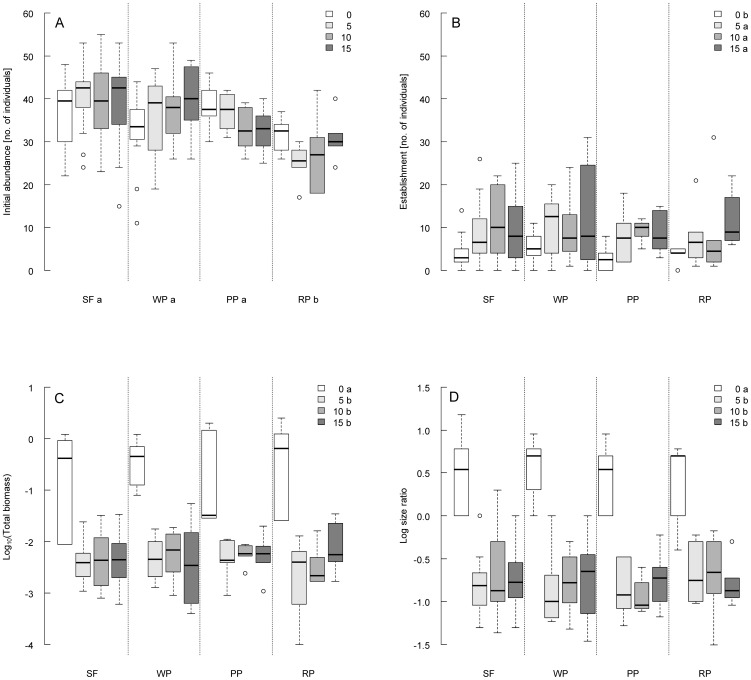
Experiment 2: Microsite availability×genetic diversity. Box and Whisker plots of A) Initial abundance (week 1), B) Establishment (week 15), C) Total biomass (given as Log_10_ for reasons of clarity, week 15) and D) Log size ratio (week 15) of *Senecio vernalis* separately by genetic diversity levels applied: SF = *seed family* (n = 18 each), WP = *within population* (n = 12 each), PP = *proximate populations* (n = 6 each), RP = *remote populations* (n = 6 each). 15, 10, 5, 0 = numbers of *Festuca* individuals as density levels applied. Different letters indicate significant differences between levels of main factors according to the Scheffé test. For further statistical details see Table 2.

Most of the variables tested displayed significant effects of *Festuca* density in the recipient matrix (Table 2). Whereas initial abundance was not significantly influenced by microsite availability, establishment after 12 weeks of the experiment, log size ratio and total biomass of *S. vernalis* were significantly affected by the treatment levels applied (Fig. 3). However, the significance was always attributable to the contrast between with and without *Festuca* in the matrix, irrespective of density of *Festuca* individuals. Establishment after 12 weeks, expressed as abundance, was significantly reduced in the control treatment (Fig. 3B). In contrast, log size ratio and total biomass, both, displayed higher values in the treatment without *Festuca*, indicating that fewer individuals survived in open soil containers, but relatively more of them reached large size classes with larger weight if compared to containers with *Festuca* as matrix (Fig. 3C, D). We found no further significant differentiation in responses towards the three *Festuca* levels for any of the variables studied (Table 2).

**Table 2:** Experiment 2: Microsite availability×genetic diversity.

Variable	Source of variation	df Num	df Den	F	p	Scheffé test
Initial abundance	Diversity	3	152	9.54	**<0.001**	SF,WP,PP>RP
[no. of individuals]	*Festuca* density	3	152	0.21	0.887	
	Diversity×*Festuca* density	9	152	1.53	0.143	
Establishment	Diversity	3	152	0.29	0.829	
[no. of individuals]	*Festuca* density	3	152	6.48	**<0.001**	15,10,5>0
	Diversity×*Festuca* density	9	152	0.43	0.917	
Log size ratio	Diversity	3	152	0.34	0.800	
	*Festuca* density	3	152	91.61	**<0.001**	0>15,10,5
	Diversity×*Festuca* density	9	152	0.35	0.958	
Total biomass	Diversity	3	135	0.31	0.815	
	*Festuca* density	3	135	89.29	**<0.001**	0>15,10,5
	Diversity×*Festuca* density	9	135	0.48	0.885	

GLM for response variables of *Senecio vernalis*. Initial abundance reflects the number of *Senecio* individuals after 1 week. Establishment refers to the number of *Senecio* individuals after 12 weeks. Abundance and total biomass were log_10_(x+1)-transformed prior to analysis. Log size ratio gives the proportion of the number of large individuals compared to those of small individuals as logarithm to the base 10. N = 168. The results of the Scheffé post hoc tests indicate the direction of significant differences between categories of each factor. 15, 10, 5, 0 indicates numbers of *Festuca* individuals as density levels applied. Seed mixtures: RP = *remote populations*, PP = *proximate populations*, WP = *within population*, SF = *seed family*. The tests of fixed effects are based on type III SS, p values and degrees of freedom of numerator (df Num) and denominator (df Den) are shown. Bold numbers indicate significant effects (p<0.05).

Repeated measures analyses of *S. vernalis* abundance displayed additional significant effects of time and time×*Festuca* density interactions (Table S6). Overall abundance of *S. vernalis* decreased with the time, but mortality was higher in open soil containers than in those with *Festuca* as matrix. Covariance analysis with calculated expected germination included as covariate into the model revealed a highly significant effect: in this model, the maximum number of individuals was exclusively affected by expected germination (Table S7).

Significant effects of genetic diversity, in contrast, were only encountered for initial abundance of *S. vernalis* in the first week (and subsequently in repeated censuses until the second week, data not shown, Table 2, Fig. 3A). Seed mixtures of remote populations yielded significantly fewer seedlings than mixtures of the other diversity levels. None of the variables studied displayed significant interactions between microsite availability and genetic diversity levels.

Presence/absence of exclusive populations in the RP mixtures displayed no additional effect (Table S8).

## Discussion

### The role of genetic diversity

Positive effects of genetic diversity on *S. vernalis* were only encountered for log size ratio in the seed density level experiment. Mixtures composed from proximate populations had a significantly higher proportion of large individuals than mixtures composed from within seed family seeds only. In addition, there was a trend of negative genetic diversity effects on initial abundance in the microsite limitation experiment. There are several explanations for the absence of strong effects of genetic diversity.

First of all, it is possible, that in the experiments, genetic diversity did not differ between the different groups composed by the populations of varying distances. Long distance dispersal might result in consistently high levels of gene flow [49], thus maintaining a high level of diversity between populations irrespective of their distance. In addition, genetic diversity might not have been decreased by using seeds from only one population or from one seed family because the species' outcrossing mating type and specialisation on insect pollination [49] counteract loss of genetic diversity among individuals. Finally, differences in genetic diversity might not have been reflected in differences in the response variables tested. Particular characteristics conferred by the seeds might have been more important than their variation (i.e. their diversity). In terms of current ecological theory, the present findings partly reflect sampling effects, thus, an increasing probability of including a particular genotype in the mixtures [14]. Consistent with our findings, most of the studies that manipulated genetic diversity in invasion biology found effects related to such genotype identity effects [14], [50]. Crawford and Whitney [14] experimentally manipulated genetic diversity of colonizing populations of *Arabidopsis thaliana* and found both complementarity among genotypes and traits associated with particular genotypes to support colonization. One of the few examples of experimental testing for effects of genetic diversity during invasions was provided by Vellend et al. [22], who studied the influence of genotype diversity of invading *Taraxacum officinale* on the invasibility of a recipient matrix composed of *Poa pratensis*. They found identity effects of the *T. officinale* genotype to be much more important for predicting invasion success than complementarity effects of genetic diversity. This result was brought about by a strong differentiation in key traits related to fitness [51].

The identity of presence or absence of populations and/or genotypes also matters for *S. vernalis*, given the strong co-varying effects of population and genotype specific germination rates encountered in one of the two experiments. This discrepancy between the strength of this covariate's effect in the two experiments was probably brought about by selecting different populations in the remote mixtures, which is also reflected in differences between their expected germination rates. Thus, mixing differently well performing seeds can result both in a positive and in a negative sampling effect, when either well or poorly performing genotypes are included just by chance. Exploring the effects of multiple introductions on invasive spread of *Centaurea stoebe*, Marrs et al. [8] could identify genetically admixed profiles in microsatellite loci that potentially contributed to the species' invasive success, thus providing evidence for positive effects of admixing genotypes at the genetic level. In contrast, Dlugosch and Parker [32] coined the term “mosaic of maladaptation” for describing negative sampling effects following multiple introductions where variation for traits can have consequences for fitness. Indeed, the levels of initially maladapted genotypes to a novel environment at the point of introduction can influence the subsequent establishment success [26], [52]. Although only experimental crossing and subsequent studies on F1/F2 generations would allow for definitely estimating genetic aspects, we can nonetheless suggest some possible explanations for direct community effects. In the present study, e.g., overall, positive and negative genotype effects were eventually offset. Experimental studies have shown that mixing negative and positive selection effects might result neither in an overall increase nor in a reduction in fitness [18], [53]. Should this explanation apply, genetic diversity would be uncoupled from performance, and in consequence, not be limiting for the *S. vernalis* invasion. However, we only performed a short-run experiment that does not allow for conclusions on fitness or for identifying selection effects. A final answer to this issue can only be provided by multi-generation experiments that exactly quantify effects on fitness and dominance of particular genotypes.

### Effects of seed density levels

As a main result of our seed density experiment, we found that seed density mattered for *S. vernalis* establishment, in particular, at the earliest stages of the life cycle, whereas at later stages of establishment, abundance was mostly reduced at the lowest seed density level. However, seed density did not affect individual performance, given that log size ratios did not respond to seed density levels nor did it affect productivity of the invasive species. This finding confirms that initial establishment of invasive species can be largely favoured by increased seed input [31], [54], (see Table 1 in [33] for an overview). Our results are consistent with the outcome of seed addition experiments summarized by Clark et al. [40] who stated that per-seed response, i.e. standardizing the response by the number of seeds added to the experimental units, was the most informative measure of seed limitation. For initial abundance data in the present seed addition experiment, per-seed responses ranged consistently between 28 and 30% for all seed density levels applied. At later stages, abundance data differed significantly between the lowest seed addition level on the one hand and the two higher levels on the other hand, resulting in per-seed responses of 19%, 16% and 13% for low, intermediate and high seed density levels, respectively. This finding, by its magnitude, is absolutely comparable to other seed sowing experiments [55]. Clark et al. [40] emphasised that in practically all studies, there is a seed-to-seedling bottleneck, brought about either by seed mortality because of decreased seed viability or abiotic and biotic factors that resulted in a mean transition rate of about 15% from added seeds to seedlings emerged. In this respect, the findings of the present study comply with the general trend; however, seem to be even higher for initial abundance data. Initial abundance very precisely reflected the numbers of the seed density levels applied. This finding is contradictory to many experiments that detected strong negative density dependence, in particular at the emergence stage, explained by increased competitive resource uptake among more similar plants [28], [56], [57]. Conversely, we did also not encounter positive density dependence, which can be attributable to facilitation in seedling establishment by reducing thermal or water stress through shading or deterring competitors and predators [58], [59]. Leger and Espeland [57] compared intra- and interspecific interactions for three native and three exotic grassland annuals and found that positive density dependence was rather species-specific and not necessarily associated with the native/exotic status of the species.

The initial high abundance of *S. vernalis* at high seed density treatments reflects the high invasion potential of this species, once seeds have arrived at a site. Nonetheless, this pattern changed for later establishment. Given that after 15 weeks the number of seedlings did not differ anymore between the intermediate and the highest seed density level indicates incipient saturation. Such an effect might hint at a shift in density-dependence across life-stages in annual plant communities [56], [60]. In their seed addition experiment with exotic *Holcus lanatus* into a Californian coastal grassland, Thomsen et al. [61] found an absolute increase in establishment with increasing number of seeds added to the plots, however both proportional initial establishment and next season survival of added seeds was highest at the lowest seed densities tested. The present study included measurements on one generation only, and more explicitly was restricted to measurements of “growth-only” stages. Therefore, we cannot make reliable predictions on variations and strengths of interactions among life stages and generations and on the temporal consistency of effects [30], [62], [63]. Similarly, increasing effects of negative density dependence during the life cycle have been described for the invasive herb *Senecio madagascariensis* [64]. For *S. vernalis*, the absence of such a trend for log size ratios (i.e. neutral density dependence) in the course of the experiment might hint at the possibility that positive and negative density effects have compensated each other although we cannot rule out the possibility that a wider range of seed density levels might have provided stronger effects. However, we tested only one beneficial environment, while the direction of density effects will become apparent only across a gradient in environmental adversity [62].

### Effects of microsite availability

We expected that the effect of seed addition of *S. vernalis* should strongly depend on the competition intensity brought about by the different *Festuca* matrix density treatments. However, in our experiment, initial abundance was completely unaffected by the treatments, thus displayed no evidence of microsite limitation. Moreover, at later stages, seedling abundance suffered from high mortality in the open soil, i.e. without *Festuca*. In contrast, these fewer individuals displayed higher individual performance and grew up to larger sizes finally resulting in increased total exotic biomass. Open soil situations, apparently, reflect more suitable sites for individual growth of *S. vernalis*, whereas initial facilitation for survival within the *Festuca* environments seems to be at the expense of competitive constraints at later stages. The sensitivity to such competitive changes during life stages, as found for *S. vernalis*, corresponds to theories on temporal shifts in plant interactions [30], [57], [64], [65]. The importance of facilitation during invasions was recently experimentally shown for *Acer negundo* seedlings by increased survival in non-*Acer* stands [66]; however, for subsequent population establishment, the authors suggest other components of interactions to be more important. In contrast, the dependence of initial *S. vernalis* abundance and performance was dependent on availability of bare ground, as provided by disturbance events. Numerous studies on invasive plant species confirm the role of disturbance on initial seedling emergence, such as the seed addition experiments with two non-native weeds of North American pastures and roadsides, *Carduus nutans* and *C. acanthoides* [67]. Similarly, it has been shown for *S. vulgaris*, congeneric to our study species, that the rate of spread was sensitive to both the size and distribution of gaps in swards [68]. In summary, our findings underline the particular ability of *S. vernalis* to colonize such gap-dominated ruderal habitat types.

## Conclusion

We found clear evidence that seed density and microsite limitation were strong determinants for invasion success of *S. vernalis* with, however, changing relative importance across subsequent early life stages. Initial abundance was mostly triggered by propagule pressure and less by microsite characteristics. Similarly, genetic diversity mattered only to a minor degree at the earliest life stages, such as for germination. However, effects of genetic diversity apparently seemed to be more decisive with regard to the probability of continued establishment, in combination with selection effects of best performing genotypes for consecutive growth. It would be promising to test for these effects on more species in replicated regions to strengthen generalization on these shifts in mechanisms. The differentiation of invasion processes into a first stage of predominant importance of propagule pressure and a second one with increasing importance of adaptation is mirrored in numerous attempts of describing the courses of invasion (e.g. [37], [69]).

## Supporting Information

Figure S1 **Locations of populations.** Distribution of populations around Halle, Germany. Populations are labelled with numbers as given in Table S1. Population 3 is hidden behind populations 4 and 5.
(TIF)

Figure S2 **Mean expected germination of** ***Senecio vernalis*** **of mixtures.** Values are provided for diversity levels *remote populations* (RP) and *proximate populations* (PP) separately by experiment. Experiment 1 = seed addition, Experiment 2 = microsite limitation, with n = 6 for each diversity level and experiment. Different letters indicate significant differences between levels of main factors according to the Scheffé test. Statistical details following ANOVA. Diversity F_1,20_ = 6.41 p = **0.020**; Experiment F_1,20_ = 0.506, p = 0.485¸ Diversity×Experiment F_1,20_ = 0.991, p = 0.331.
(TIF)

Table S1 **Location and habitat type affiliation of sampled** ***S. vernalis*** **populations in Eastern Germany.**
(DOC)

Table S2 **Details on exact composition of seed mixtures of** ***S. vernalis*** **in the four diversity levels applied by population and individual identity in the two container experiments.** Ind shows the identity of seed families, “-” indicates pooled samples of a population without exact knowledge of seed family identity. Pop indicates the identity of the population referred to in the mixtures. All mixtures were exactly replicated for application to different seed density levels (Experiment 1) and *Festuca rupicola* density levels (Experiment 2), respectively. Potential germination capacities give the expected germination for populations and seed families determined in a previous study (Hantsch et al., unpublished). Potential germination capacities of a particular seed mixture applied were calculated as the mean germination capacities across the populations involved in the respective mixture.
(DOC)

Table S3 **Experiment 1: Propagule pressure×genetic diversity.** Repeated measures GLM for number of individuals *Senecio vernalis* with fixed factors diversity, seed density and time. N = 126. The tests of fixed effects are based on type III SS, p values and degrees of freedom of numerator (df Num) and denominator (df Den) are shown. Bold p values indicate significant effects (p<0.05).
(DOC)

Table S4 **Experiment 1: Propagule pressure×genetic diversity.** GLM for maximum abundance of *Senecio vernalis*, including expected germination as covariate in the model. N = 126. The tests of fixed effects are based on type III SS, p values and degrees of freedom of numerator (df Num) and denominator (df Den) are shown. Bold numbers indicate significant effects (p<0.05).
(DOC)

Table S5 **Experiment 1: Propagule pressure×genetic diversity and population identity effects.** GLM for response variables of *Senecio vernalis* testing for presence/absence effects of those populations (pop01, pop05, pop13) exclusively assigned to the highest diversity level (RP = *remote populations*) in this experiment only. Initial abundance reflects the number of *Senecio* individuals after 1 week. Establishment refers to the number of *Senecio* individuals after 15 weeks. Abundance and total biomass were log_10_(x+1)-transformed prior to analysis. Log size ratio gives the proportion of number of large individuals compared to those of small individuals as logarithm to the base 10. N = 126. The results of the Scheffé post hoc tests indicate the direction of significant differences between categories of each factor. 90, 60, 30 indicates seed density levels applied. The tests of fixed effects are based on type III SS, p values and degrees of freedom of numerator (df Num) and denominator (df Den) are shown. Bold numbers indicate significant effects (p<0.05).
(DOC)

Table S6 **Experiment 2: Microsite availability×genetic diversity.** GLM for number of individuals, including time as additional fixed factor in the model. The tests of fixed effects are based on type III SS, p values and degrees of freedom of numerator (df Num) and denominator (df Den) are shown. Bold numbers indicate significant effects (p<0.05).
(DOC)

Table S7 **Experiment 2: Microsite availability×genetic diversity.** GLM for maximum abundance of *Senecio vernalis*, including expected germination as covariate in the model. N = 168. The tests of fixed effects are based on type III SS, p values and degrees of freedom of numerator (df Num) and denominator (df Den) are shown. Bold numbers indicate significant effects (p<0.05). GLM for maximum abundance of *Senecio vernalis*, including expected germination as covariate in the model. N = 168. The tests of fixed effects are based on type III SS, p values and degrees of freedom of numerator (df Num) and denominator (df Den) are shown. Bold numbers indicate significant effects (p<0.05).
(DOC)

Table S8 **Experiment 2: Microsite availability×genetic diversity and population identity effects.** GLM for response variables of *Senecio vernalis* testing for presence/absence effects of those populations (pop03, pop04, pop06) exclusively assigned to the highest diversity level (RP = *remote populations*) in this experiment only. Initial abundance reflects the number of *Senecio* individuals after 1 week. Establishment refers to the number of *Senecio* individuals after 12 weeks. Abundance and total biomass were log_10_(x+1)-transformed prior to analysis. Log size ratio gives the proportion of the number of large individuals compared to those of small individuals as logarithm to the base 10. N = 168. The results of the Scheffé post hoc tests indicate the direction of significant differences between categories of each factor. 15, 10, 5, 0 indicates numbers of *Festuca* individuals as density levels applied. Seed mixtures: RP = *remote populations*, PP = *proximate populations*, WP = *within population*, SF = *seed family*. The tests of fixed effects are based on type III SS, p values and degrees of freedom of numerator (df Num) and denominator (df Den) are shown. Bold numbers indicate significant effects (p<0.05).
(DOC)
