# Supplementary figures and images for: The Role of Propagule Pressure, Genetic Diversity and Microsite Availability for Senecio vernalis Invasion
Source: PLoS One. 2013 Feb 20;8(2):e57029. doi: 10.1371/journal.pone.0057029 (PMC3577778; doi:10.1371/journal.pone.0057029)

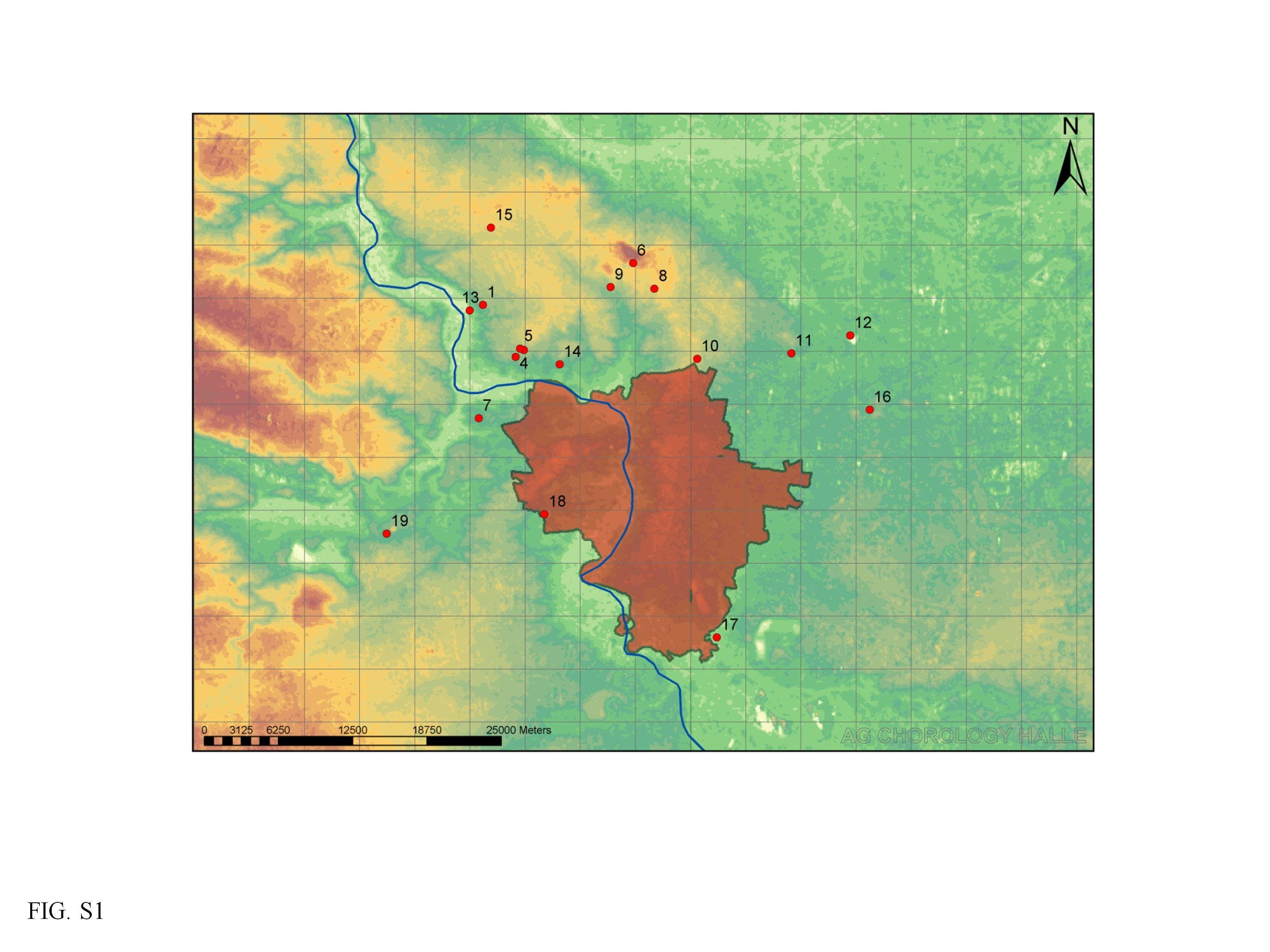

Supplement: Figure S1 — Locations of populations. Distribution of populations around Halle, Germany. Populations are labelled with numbers as given in Table S1. Population 3 is hidden behind populations 4 and 5. (TIF) [file pone.0057029.s001.tif]
