# Supplementary material for: The Role of Propagule Pressure, Genetic Diversity and Microsite Availability for Senecio vernalis Invasion
Source: PLoS One. 2013 Feb 20;8(2):e57029. doi: 10.1371/journal.pone.0057029 (PMC3577778; doi:10.1371/journal.pone.0057029)
Supplement: Table S1 — Location and habitat type affiliation of sampled S. vernalis populations in Eastern Germany. (DOC) [file pone.0057029.s003.doc]

**Table S1.** **Location and habitat type affiliation of sampled *S. vernalis* populations in Eastern Germany.**

| Population | Latitude [°N] | Longitude [°E] | Habitat type | Population area [m x m] | Estimated population size [number of individuals] |
| --- | --- | --- | --- | --- | --- |
|
| 1 | 51.57192 | 11.84336 | Semi-dry grassland | 40 x 8 | 100 |
| 2 | 51.05979 | 14.40188 | Railway track | 70 x 3 | 50 |
| 3 | 51.55074 | 11.87424 | Boundary margin | 30 x 2 | 100 |
| 4 | 51.54736 | 11.86814 | Boundary margin | 30 x 2 | 50 |
| 5 | 51.55126 | 11.87139 | Boundary margin | 25 x 1.5 | 50 |
| 6 | 51.59164 | 11.95678 | Soil dump | 8 x 7 | 30 |
| 7 | 51.52487 | 11.84295 | Gravel quarry | 100 x 70 | 100 |
| 8 | 51.57955 | 11.97288 | Sand quarry | 20 x 2 | 50 |
| 9 | 51.58038 | 11.93974 | Sand quarry | 200 x 200 | 50 |
| 10 | 51.54658 | 12.00519 | Gravel quarry | 250 x 150 | 75 |
| 11 | 51.54909 | 12.07626 | Semi-dry grassland | 50 x 40 | 35 |
| 12 | 51.55762 | 12.12077 | Roadside | 45 x 2 | 40 |
| 13 | 51.59793 | 11.83129 | Grassland | 190 x 3 | 35 |
| 14 | 51.54403 | 11.90130 | Clay quarry | 50 x 4 | 40 |
| 15 | 51.60826 | 11.84944 | Roadside | 30 x 1 | 30 |
| 16 | 51.52249 | 12.13550 | Roadside | 200 x 3 | 30 |
| 17 | 51.41513 | 12.02009 | Roadside | 150 x 1.5 | 40 |
| 18 | 51.47325 | 11.88970 | Stone quarry | 50 x 5 | 10 |
| 19 | 51.46412 | 11.77055 | Semi-dry grassland | 8 x 4 | 20 |
