# Supplementary material for: The Role of Propagule Pressure, Genetic Diversity and Microsite Availability for Senecio vernalis Invasion
Source: PLoS One. 2013 Feb 20;8(2):e57029. doi: 10.1371/journal.pone.0057029 (PMC3577778; doi:10.1371/journal.pone.0057029)
Supplement: Table S2 — Details on exact composition of seed mixtures of S. vernalis in the four diversity levels applied by population and individual identity in the two container experiments. Ind shows the identity of seed families, “-” indicates pooled samples of a population without exact knowledge of seed family identity. Pop indicates the identity of the population referred to in the mixtures. All mixtures were exactly replicated for application to different seed density levels (Experiment 1) and Festuca rupicola density levels (Experiment 2), respectively. Potential germination capacities give the expected germination for populations and seed families determined in a previous study (Hantsch et al., unpublished). Potential germination capacities of a particular seed mixture applied were calculated as the mean germination capacities across the populations involved in the respective mixture. (DOC) [file pone.0057029.s004.doc]

**Table S2.** **Details on exact composition of seed mixtures of *S. vernalis* in the four diversity levels applied by population and individual identity in the two container experiments.**

|  |  | **Experiment 1: Propagule pressure x genetic diversity** | | | | | | | | | |  | **Experiment 2: Microsite availability x genetic diversity** | | | | | | | |
| --- | --- | --- | --- | --- | --- | --- | --- | --- | --- | --- | --- | --- | --- | --- | --- | --- | --- | --- | --- | --- |
| Diversity level | Replicate | Ind | Pop1 | Pop2 | Pop3 | Pop4 | Pop5 | number of seeds by Pop/Ind | | | Potential germination capacity |  | Ind | Pop1 | Pop2 | Pop3 | Pop4 | Pop5 | number of seeds by Pop/Ind | Potential germination capacity |
|  |  |  |  |  |  |  |  | Density level | | | [%] |  |  |  |  |  |  |  | Density level | [%] |
|  |  |  |  |  |  |  |  | 30 | 60 | 90 |  |  |  |  |  |  |  |  | 60 |  |
| *RP - Remote populations* | | |  |  |  |  |  |  |  |  |  |  |  |  |  |  |  |  |  |  |
|  | 1 | - | 1 | 5 | 8 | 11 | 17 | 6 | 12 | 18 | 89.3 |  | - | 4 | 6 | 7 | 11 | 17 | 12 | 71.1 |
|  | 2 | - | 7 | 10 | 11 | 15 | 19 | 6 | 12 | 18 | 77.0 |  | - | 7 | 8 | 12 | 17 | 19 | 12 | 69.7 |
|  | 3 | - | 9 | 11 | 13 | 15 | 17 | 6 | 12 | 18 | 87.0 |  | - | 3 | 6 | 8 | 11 | 17 | 12 | 73.6 |
|  | 4 | - | 12 | 14 | 15 | 16 | 19 | 6 | 12 | 18 | 87.0 |  | - | 10 | 14 | 16 | 17 | 19 | 12 | 60.6 |
|  | 5 | - | 7 | 8 | 9 | 16 | 19 | 6 | 12 | 18 | 83.0 |  | - | 7 | 8 | 11 | 15 | 17 | 12 | 71.9 |
|  | 6 | - | 5 | 10 | 12 | 16 | 17 | 6 | 12 | 18 | 79.3 |  | - | 7 | 9 | 12 | 17 | 19 | 12 | 83.0 |
| *PP - Proximate populations* | | | |  |  |  |  |  |  |  |  |  |  |  |  |  |  |  |  |  |
|  | 7 | - | 3 | 4 | 5 | 9 | 10 | 6 | 12 | 18 | 84.3 |  | - | 3 | 4 | 5 | 9 | 14 | 12 | 94.0 |
|  | 8 | - | 3 | 4 | 6 | 8 | 10 | 6 | 12 | 18 | 84.7 |  | - | 3 | 4 | 9 | 10 | 14 | 12 | 84.7 |
|  | 9 | - | 4 | 5 | 8 | 10 | 14 | 6 | 12 | 18 | 85.7 |  | - | 3 | 4 | 8 | 10 | 14 | 12 | 85.3 |
|  | 10 | - | 3 | 4 | 6 | 10 | 14 | 6 | 12 | 18 | 86.0 |  | - | 3 | 5 | 8 | 10 | 14 | 12 | 73.3 |
|  | 11 | - | 4 | 6 | 8 | 10 | 14 | 6 | 12 | 18 | 85.3 |  | - | 3 | 5 | 8 | 9 | 14 | 12 | 95.7 |
|  | 12 | - | 4 | 5 | 6 | 8 | 14 | 6 | 12 | 18 | 94.7 |  | - | 3 | 4 | 8 | 9 | 14 | 12 | 93.0 |
| *WP - Population* | |  |  |  |  |  |  |  |  |  |  |  |  |  |  |  |  |  |  |  |
|  | 13 | - | 1 | - | - | - | - | 30 | 60 | 90 | 100.0 |  | - | 12 | - | - | - | - | 60 | 100.0 |
|  | 14 | - | 8 | - | - | - | - | 30 | 60 | 90 | 93.3 |  | - | 11 | - | - | - | - | 60 | 85.0 |
|  | 15 | - | 10 | - | - | - | - | 30 | 60 | 90 | 51.7 |  | - | 16 | - | - | - | - | 60 | 76.7 |
|  | 16 | - | 12 | - | - | - | - | 30 | 60 | 90 | 100.0 |  | - | 6 | - | - | - | - | 60 | 96.7 |
|  | 17 | - | 13 | - | - | - | - | 30 | 60 | 90 | 96.7 |  | - | 17 | - | - | - | - | 60 | 70.0 |
|  | 18 | - | 17 | - | - | - | - | 30 | 60 | 90 | 70.0 |  | - | 7 | - | - | - | - | 60 | 90.0 |
|  | 19 | - | 3 | - | - | - | - | 30 | 60 | 90 | 96.7 |  | - | 5 | - | - | - | - | 60 | 98.3 |
|  | 20 | - | 4 | - | - | - | - | 30 | 60 | 90 | 85.0 |  | - | 4 | - | - | - | - | 60 | 85.0 |
|  | 21 | - | 5 | - | - | - | - | 30 | 60 | 90 | 98.3 |  | - | 8 | - | - | - | - | 60 | 93.3 |
|  | 22 | - | 7 | - | - | - | - | 30 | 60 | 90 | 90.0 |  | - | 3 | - | - | - | - | 60 | 96.7 |
|  | 23 | - | 9 | - | - | - | - | 30 | 60 | 90 | 90.0 |  | - | 14 | - | - | - | - | 60 | 100.0 |
|  | 24 | - | 14 | - | - | - | - | 30 | 60 | 90 | 100.0 |  | - | 9 | - | - | - | - | 60 | 90.0 |
| *SF - Seed family* | |  |  |  |  |  |  |  |  |  |  |  |  |  |  |  |  |  |  |  |
|  | 25 | 14 | 1 | - | - | - | - | 30 | 60 | 90 | 100.0 |  | 8 | 6 | - | - | - | - | 60 | 91.7 |
|  | 26 | 19 | 1 | - | - | - | - | 30 | 60 | 90 | 100.0 |  | 17 | 6 | - | - | - | - | 60 | 100.0 |
|  | 27 | 4 | 1 | - | - | - | - | 30 | 60 | 90 | 100.0 |  | 9 | 6 | - | - | - | - | 60 | 96.7 |
|  | 28 | 2 | 8 | - | - | - | - | 30 | 60 | 90 | 96.7 |  | 14 | 11 | - | - | - | - | 60 | 96.7 |
|  | 29 | 16 | 8 | - | - | - | - | 30 | 60 | 90 | 98.3 |  | 1 | 11 | - | - | - | - | 60 | 100.0 |
|  | 30 | 4 | 8 | - | - | - | - | 30 | 60 | 90 | 98.3 |  | 2 | 11 | - | - | - | - | 60 | 85.0 |
|  | 31 | 20 | 12 | - | - | - | - | 30 | 60 | 90 | 98.3 |  | 18 | 12 | - | - | - | - | 60 | 98.3 |
|  | 32 | 14 | 12 | - | - | - | - | 30 | 60 | 90 | 100.0 |  | 6 | 12 | - | - | - | - | 60 | 95.0 |
|  | 33 | 8 | 12 | - | - | - | - | 30 | 60 | 90 | 100.0 |  | 10 | 12 | - | - | - | - | 60 | 95.0 |
|  | 34 | 16 | 14 | - | - | - | - | 30 | 60 | 90 | 96.7 |  | 4 | 5 | - | - | - | - | 60 | 98.3 |
|  | 35 | 17 | 14 | - | - | - | - | 30 | 60 | 90 | 93.3 |  | 10 | 5 | - | - | - | - | 60 | 98.3 |
|  | 36 | 7 | 14 | - | - | - | - | 30 | 60 | 90 | 96.7 |  | 8 | 5 | - | - | - | - | 60 | 100.0 |
|  | 37 | 4 | 3 | - | - | - | - | 30 | 60 | 90 | 95.0 |  | 19 | 14 | - | - | - | - | 60 | 90.0 |
|  | 38 | 2 | 3 | - | - | - | - | 30 | 60 | 90 | 100.0 |  | 1 | 14 | - | - | - | - | 60 | 98.3 |
|  | 39 | 21 | 3 | - | - | - | - | 30 | 60 | 90 | 97.2 |  | 6 | 14 | - | - | - | - | 60 | 90.0 |
|  | 40 | 3 | 5 | - | - | - | - | 30 | 60 | 90 | 100.0 |  | 13 | 3 | - | - | - | - | 60 | 96.7 |
|  | 41 | 2 | 5 | - | - | - | - | 30 | 60 | 90 | 91.7 |  | 9 | 3 | - | - | - | - | 60 | 100.0 |
|  | 42 | 4 | 5 | - | - | - | - | 30 | 60 | 90 | 98.3 |  | 11 | 3 | - | - | - | - | 60 | 96.7 |

Ind shows the identity of seed families, "-" indicates pooled samples of a population without exact knowledge of seed family identity. Pop indicates the identity of the population referred to in the mixtures. All mixtures were exactly replicated for application to different seed density levels (Experiment 1) and *Festuca rupicola* density levels (Experiment 2), respectively. Potential germination capacities give the expected germination for populations and seed families determined in a previous study (Hantsch et al., unpublished). Potential germination capacities of a particular seed mixture applied were calculated as the mean germination capacities across the populations involved in the respective mixture.
