# Supplementary material for: The Role of Propagule Pressure, Genetic Diversity and Microsite Availability for Senecio vernalis Invasion
Source: PLoS One. 2013 Feb 20;8(2):e57029. doi: 10.1371/journal.pone.0057029 (PMC3577778; doi:10.1371/journal.pone.0057029)
Supplement: Table S3 — Experiment 1: Propagule pressure×genetic diversity. Repeated measures GLM for number of individuals Senecio vernalis with fixed factors diversity, seed density and time. N = 126. The tests of fixed effects are based on type III SS, p values and degrees of freedom of numerator (df Num) and denominator (df Den) are shown. Bold p values indicate significant effects (p<0.05). (DOC) [file pone.0057029.s005.doc]

**Table S3.** **Experiment 1: Propagule pressure x genetic diversity.**

| Source of variation | df Num | df Den | F | p |
| --- | --- | --- | --- | --- |
| Diversity | 3 | 114 | 1.42 | 0.242 |
| Seed density | 2 | 114 | 98.4 | **<0.001** |
| Diversity x seed density | 6 | 114 | 1.18 | 0.323 |
| Time | 8 | 984 | 1628.8 | **<0.001** |
| Time x seed density | 16 | 984 | 18.53 | **<0.001** |

Repeated measures GLM for number of individuals *Senecio vernalis* with fixed factors diversity, seed density and time. N = 126. The tests of fixed effects are based on type III SS, p values and degrees of freedom of numerator (df Num) and denominator (df Den) are shown. Bold p values indicate significant effects (p < 0.05).
