# Supplementary material for: The Role of Propagule Pressure, Genetic Diversity and Microsite Availability for Senecio vernalis Invasion
Source: PLoS One. 2013 Feb 20;8(2):e57029. doi: 10.1371/journal.pone.0057029 (PMC3577778; doi:10.1371/journal.pone.0057029)
Supplement: Table S4 — Experiment 1: Propagule pressure×genetic diversity. GLM for maximum abundance of Senecio vernalis, including expected germination as covariate in the model. N = 126. The tests of fixed effects are based on type III SS, p values and degrees of freedom of numerator (df Num) and denominator (df Den) are shown. Bold numbers indicate significant effects (p<0.05). (DOC) [file pone.0057029.s006.doc]

**Table S4.** **Experiment 1: Propagule pressure x genetic diversity.**

| Source of variation | df Num | df Den | F | p |
| --- | --- | --- | --- | --- |
| Diversity | 3 | 113 | 0.42 | 0.741 |
| Seed density | 2 | 113 | 120.7 | **<0.001** |
| Diversity x seed density | 6 | 113 | 1.84 | 0.097 |
| Germination | 1 | 113 | 0.69 | 0.407 |

GLM for maximum abundance of *Senecio vernalis*, including expected germination as covariate in the model. N = 126. The tests of fixed effects are based on type III SS, p values and degrees of freedom of numerator (df Num) and denominator (df Den) are shown. Bold numbers indicate significant effects (p < 0.05).
