# Supplementary material for: The Role of Propagule Pressure, Genetic Diversity and Microsite Availability for Senecio vernalis Invasion
Source: PLoS One. 2013 Feb 20;8(2):e57029. doi: 10.1371/journal.pone.0057029 (PMC3577778; doi:10.1371/journal.pone.0057029)
Supplement: Table S5 — Experiment 1: Propagule pressure×genetic diversity and population identity effects. GLM for response variables of Senecio vernalis testing for presence/absence effects of those populations (pop01, pop05, pop13) exclusively assigned to the highest diversity level (RP = remote populations) in this experiment only. Initial abundance reflects the number of Senecio individuals after 1 week. Establishment refers to the number of Senecio individuals after 15 weeks. Abundance and total biomass were log10(x+1)-transformed prior to analysis. Log size ratio gives the proportion of number of large individuals compared to those of small individuals as logarithm to the base 10. N = 126. The results of the Scheffé post hoc tests indicate the direction of significant differences between categories of each factor. 90, 60, 30 indicates seed density levels applied. The tests of fixed effects are based on type III SS, p values and degrees of freedom of numerator (df Num) and denominator (df Den) are shown. Bold numbers indicate significant effects (p<0.05). (DOC) [file pone.0057029.s007.doc]

**Table S5. Experiment 1:** Propagule pressure x genetic diversity and population identity effects

| Variable | Source of variation | df Num | df Den | F | p | Scheffe test |
| --- | --- | --- | --- | --- | --- | --- |
| Initial abundance | Diversity | 3 | 111 | 1.7 | 0.170 |  |
| [no. of individuals] | Seed density | 2 | 111 | 60.58 | **<0.001** | 90>60>30 |
|  | Diversity x seed density | 6 | 111 | 0.99 | 0.434 |  |
|  | pop01 | 1 | 111 | 0.44 | 0.507 |  |
|  | pop05 | 1 | 111 | 6.72 | **0.011** |  |
|  | pop13 | 1 | 111 | 3.08 | 0.082 |  |
|  |  |  |  |  |  |  |
| Establishment | Diversity | 3 | 114 | 1.23 | 0.302 |  |
| [no. of individuals] | Seed density | 2 | 114 | 14.37 | **<0.001** | 90,60>30 |
|  | Diversity x seed density | 6 | 114 | 0.63 | 0.703 |  |
|  | pop01 | 1 | 111 | 0.13 | 0.720 |  |
|  | pop05 | 1 | 111 | 8.47 | **0.004** |  |
|  | pop13 | 1 | 111 | 1.73 | 0.192 |  |
|  |  |  |  |  |  |  |
| Log size ratio | Diversity | 3 | 111 | 2.48 | 0.065 |  |
|  | Seed density | 2 | 111 | 2.58 | 0.080 |  |
|  | Diversity x seed density | 6 | 111 | 1.99 | 0.072 |  |
|  | pop01 | 1 | 111 | 8.31 | **0.005** |  |
|  | pop05 | 1 | 111 | 1.53 | 0.218 |  |
|  | pop13 | 1 | 111 | 0.25 | 0.616 |  |
|  |  |  |  |  |  |  |
| Total biomass | Diversity | 3 | 107 | 2.07 | 0.108 |  |
|  | Seed density | 2 | 107 | 3.02 | 0.053 |  |
|  | Diversity x seed density | 6 | 107 | 2.01 | 0.071 |  |
|  | pop01 | 1 | 107 | 3.67 | 0.058 |  |
|  | pop05 | 1 | 107 | 1.66 | 0.201 |  |
|  | pop13 | 1 | 107 | 5.35 | **0.023** |  |

GLM for response variables of *Senecio vernalis* testing for presence/absence effects of those populations (pop01, pop05, pop13) exclusively assigned to the highest diversity level (RP = *remote populations*) in this experiment only. Initial abundance reflects the number of *Senecio* individuals after 1 week. Establishment refers to the number of *Senecio* individuals after 15 weeks. Abundance and total biomass were log_10_(x+1)-transformed prior to analysis. Log size ratio gives the proportion of number of large individuals compared to those of small individuals as logarithm to the base 10. N = 126. The results of the Scheffé post hoc tests indicate the direction of significant differences between categories of each factor. 90, 60, 30 indicates seed density levels applied. The tests of fixed effects are based on type III SS, p values and degrees of freedom of numerator (df Num) and denominator (df Den) are shown. Bold numbers indicate significant effects (p < 0.05) .
