# Supplementary material for: The Role of Propagule Pressure, Genetic Diversity and Microsite Availability for Senecio vernalis Invasion
Source: PLoS One. 2013 Feb 20;8(2):e57029. doi: 10.1371/journal.pone.0057029 (PMC3577778; doi:10.1371/journal.pone.0057029)
Supplement: Table S6 — Experiment 2: Microsite availability×genetic diversity. GLM for number of individuals, including time as additional fixed factor in the model. The tests of fixed effects are based on type III SS, p values and degrees of freedom of numerator (df Num) and denominator (df Den) are shown. Bold numbers indicate significant effects (p<0.05). (DOC) [file pone.0057029.s008.doc]

**Table S6.** **Experiment 2: Microsite availability x genetic diversity.**

| Source of variation | df Num | df Den | F | p |
| --- | --- | --- | --- | --- |
| Diversity | 3 | 152 | 0.83 | 0.481 |
| *Festuca* densit*y* | 3 | 152 | 13.5 | **<0.001** |
| Diversity x *Festuca* density | 9 | 152 | 0.31 | 0.9702 |
| Time | 7 | 1148 | 1936.42 | **<0.001** |
| Time x *Festuca* density | 21 | 1148 | 6.68 | **<0.001** |

GLM for number of individuals, including time as additional fixed factor in the model. The tests of fixed effects are based on type III SS, p values and degrees of freedom of numerator (df Num) and denominator (df Den) are shown. Bold numbers indicate significant effects (p < 0.05).
