# Supplementary material for: The Role of Propagule Pressure, Genetic Diversity and Microsite Availability for Senecio vernalis Invasion
Source: PLoS One. 2013 Feb 20;8(2):e57029. doi: 10.1371/journal.pone.0057029 (PMC3577778; doi:10.1371/journal.pone.0057029)
Supplement: Table S7 — Experiment 2: Microsite availability×genetic diversity. GLM for maximum abundance of Senecio vernalis, including expected germination as covariate in the model. N = 168. The tests of fixed effects are based on type III SS, p values and degrees of freedom of numerator (df Num) and denominator (df Den) are shown. Bold numbers indicate significant effects (p<0.05). GLM for maximum abundance of Senecio vernalis, including expected germination as covariate in the model. N = 168. The tests of fixed effects are based on type III SS, p values and degrees of freedom of numerator (df Num) and denominator (df Den) are shown. Bold numbers indicate significant effects (p<0.05). (DOC) [file pone.0057029.s009.doc]

**Table S7.** **Experiment 2: Microsite availability x genetic diversity.** GLM for maximum abundance of *Senecio vernalis*, including expected germination as covariate in the model. N = 168. The tests of fixed effects are based on type III SS, p values and degrees of freedom of numerator (df Num) and denominator (df Den) are shown. Bold numbers indicate significant effects (p < 0.05).

| Source of variation | df Num | df Den | F | p |
| --- | --- | --- | --- | --- |
| Diversity | 3 | 151 | 0.22 | 0.884 |
| *Festuca* density | 2 | 151 | 0.71 | 0.549 |
| Diversity x *Festuca* density | 6 | 151 | 1.20 | 0.302 |
| Germination | 1 | 151 | 37.31 | **<0.001** |

GLM for maximum abundance of *Senecio vernalis*, including expected germination as covariate in the model. N = 168. The tests of fixed effects are based on type III SS, p values and degrees of freedom of numerator (df Num) and denominator (df Den) are shown. Bold numbers indicate significant effects (p < 0.05).
