# Supplementary material for: The Role of Propagule Pressure, Genetic Diversity and Microsite Availability for Senecio vernalis Invasion
Source: PLoS One. 2013 Feb 20;8(2):e57029. doi: 10.1371/journal.pone.0057029 (PMC3577778; doi:10.1371/journal.pone.0057029)
Supplement: Table S8 — Experiment 2: Microsite availability×genetic diversity and population identity effects. GLM for response variables of Senecio vernalis testing for presence/absence effects of those populations (pop03, pop04, pop06) exclusively assigned to the highest diversity level (RP = remote populations) in this experiment only. Initial abundance reflects the number of Senecio individuals after 1 week. Establishment refers to the number of Senecio individuals after 12 weeks. Abundance and total biomass were log10(x+1)-transformed prior to analysis. Log size ratio gives the proportion of the number of large individuals compared to those of small individuals as logarithm to the base 10. N = 168. The results of the Scheffé post hoc tests indicate the direction of significant differences between categories of each factor. 15, 10, 5, 0 indicates numbers of Festuca individuals as density levels applied. Seed mixtures: RP = remote populations, PP = proximate populations, WP = within population, SF = seed family. The tests of fixed effects are based on type III SS, p values and degrees of freedom of numerator (df Num) and denominator (df Den) are shown. Bold numbers indicate significant effects (p<0.05). (DOC) [file pone.0057029.s010.doc]

**Table S8. Experiment 2:** Microsite availability x genetic diversity and population identity effects

| Variable | Source of variation | df Num | df Den | F | p | Scheffe test |
| --- | --- | --- | --- | --- | --- | --- |
| Initial abundance | Diversity | 3 | 149 | 12.08 | **<0.001** | SF,WP,(PP>RP) |
| [no. of individuals] | *Festuca density* | 3 | 149 | 0.23 | 0.875 |  |
|  | Diversity x Festuca density | 9 | 149 | 1.65 | 0.105 |  |
|  | pop03 | 1 | 149 | 5.76 | 0.018 |  |
|  | pop04 | 1 | 149 | 0.64 | 0.424 |  |
|  | pop06 | 1 | 149 | 6.19 | 0.014 |  |
|  |  |  |  |  |  |  |
| Establishment | Diversity | 3 | 149 | 0.49 | 0.693 |  |
| [no. of individuals] | *Festuca density* | 3 | 149 | 6.50 | **<0.001** | 15,10,5>0 |
|  | Diversity x Festuca density | 9 | 149 | 0.43 | 0.916 |  |
|  | pop03 | 1 | 149 | 0.00 | 0.971 |  |
|  | pop04 | 1 | 149 | 1.04 | 0.310 |  |
|  | pop06 | 1 | 149 | 1.38 | 0.241 |  |
|  |  |  |  |  |  |  |
| Log size ratio | Diversity | 3 | 149 | 0.48 | 0.700 |  |
|  | *Festuca density* | 3 | 149 | 90.84 | **<0.001** | 0>15,10,5 |
|  | Diversity x Festuca density | 9 | 149 | 0.34 | 0.959 |  |
|  | pop03 | 1 | 149 | 0.39 | 0.536 |  |
|  | pop04 | 1 | 149 | 0.01 | 0.916 |  |
|  | pop06 | 1 | 149 | 1.16 | 0.282 |  |
|  |  |  |  |  |  |  |
| Total biomass | Diversity | 3 | 132 | 0.75 | 0.525 |  |
|  | *Festuca density* | 3 | 132 | 89.44 | **<0.001** | 0>15,10,5 |
|  | *Diversity x Festuca density* | 9 | 132 | 0.46 | 0.898 |  |
|  | pop03 | 1 | 132 | 0.45 | 0.503 |  |
|  | pop04 | 1 | 132 | 0.26 | 0.610 |  |
|  | pop06 | 1 | 132 | 1.58 | 0.211 |  |

GLM for response variables of *Senecio vernalis* testing for presence/absence effects of those populations (pop03, pop04, pop06) exclusively assigned to the highest diversity level (RP = *remote populations*) in this experiment only. Initial abundance reflects the number of *Senecio* individuals after 1 week. Establishment refers to the number of *Senecio* individuals after 12 weeks. Abundance and total biomass were log_10_(x+1)-transformed prior to analysis. Log size ratio gives the proportion of the number of large individuals compared to those of small individuals as logarithm to the base 10. N = 168. The results of the Scheffé post hoc tests indicate the direction of significant differences between categories of each factor. 15, 10, 5, 0 indicates numbers of *Festuca* individuals as density levels applied. Seed mixtures: RP = *remote populations*, PP = *proximate populations*, WP = *within population*, SF = *seed family*. The tests of fixed effects are based on type III SS, p values and degrees of freedom of numerator (df Num) and denominator (df Den) are shown. Bold numbers indicate significant effects (p < 0.05).
